# BILP-19—An Ultramicroporous Organic Network with Exceptional Carbon Dioxide Uptake

**DOI:** 10.3390/molecules22081343

**Published:** 2017-08-12

**Authors:** Christoph Klumpen, Florian Radakovitsch, Andreas Jess, Jürgen Senker

**Affiliations:** 1 Inorganic Chemistry III, University of Bayreuth, Universitätsstraße 30, 95440 Bayreuth, Germany; christoph.klumpen@uni-bayreuth.de; 2 Chair of Chemical Engineering, University of Bayreuth, Universitätsstraße 30, 95440 Bayreuth, Germany; florian.radakovitsch@uni-bayreuth.de (F.R.); jess@uni-bayreuth.de (A.J.)

**Keywords:** Benzimidazole linked polymers, gas-sorption, carbon dioxide adsorption, methane adsorption, water vapor sorption, carbon dioxide capture and storage, microporous organic polymers

## Abstract

Porous benzimidazole-based polymers (BILPs) have proven to be promising for carbon dioxide capture and storage. The polarity of their chemical structure in combination with an inherent porosity allows for adsorbing large amounts of carbon dioxide in combination with high selectivities over unpolar guest molecules such as methane and nitrogen. For this reason, among purely organic polymers, BILPs contain some of the most effective networks to date. Nevertheless, they are still outperformed by competitive materials such as metal-organic frameworks (MOFs) or metal doped porous polymers. Here, we report the synthesis of BILP-19 and its exceptional carbon dioxide uptake of up to 6 mmol·g^−1^ at 273 K, making the network comparable to state-of-the-art materials. BILP-19 precipitates in a particulate structure with a strongly anisotropic growth into platelets, indicating a sheet-like structure for the network. It exhibits only a small microporous but a remarkable ultra-microporous surface area of 144 m^2^·g^−1^ and 1325 m^2^·g^−1^, respectively. We attribute the exceptional uptake of small guest molecules such as carbon dioxide and water to the distinct ultra-microporosity. Additionally, a pronounced hysteresis for both guests is observed, which in combination with the platelet character is probably caused by an expansion of the interparticle space, creating additional accessible ultra-microporous pore volume. For nitrogen and methane, this effect does not occur which explains their low affinity. In consequence, Henry selectivities of 123 for CO_2_/N_2_ at 298 K and 12 for CO_2_/CH_4_ at 273 K were determined. The network was carefully characterized with solid-state nuclear magnetic resonance (NMR) and infrared (IR) spectroscopy, thermal gravimetry (TG) and elemental analyses as well as physisorption experiments with Ar, N_2_, CO_2_, CH_4_ and water.

## 1. Introduction

The reduction of anthropogenic carbon dioxide emission as main contributor of global warming is a crucial task of today’s society. Therefore, in 2015, within the Paris Agreement, 195 states agreed to reduce the risks and impacts of climate change [1]. According to this, short term technologies such as carbon capture and storage (CCS) are needed to ensure effective and cost efficient filtration of industrial flue gas emissions [2]. CCS technologies combine both the selective capture of CO_2_ and its energetically beneficious storage. In this respect, adsorptive materials such as amines, alkalized alumina or carbonates already play a key role, especially in post- and precombustion filtration. However, the major drawback of those materials is their chemisorptive interaction with once captured carbon dioxide, which leads to high regeneration. In this respect, physisorptive materials have gained attention, due to their applicability for highly efficient approaches such as thermal- or pressure swing adsorption [3]. Furthermore, easier access to the once captured gas is the key to subsequent carbon dioxide-based chemistry. Additionally, a high physical and chemical stability is often required to ensure long-term stability and utilizability upon operation. In this respect, microporous organic polymers (MOPs) have proven to be a promising class of materials for effective carbon dioxide adsorption [4,5]. Their lightweight morphology, quasi-unlimited diversity and high physicochemical stability are the main advantages of MOPs. Thus, many different types of networks with partially excellent performances have been investigated in the past decade, showing that for a high carbon dioxide uptake the content of ultra-micropores (<0.7 nm), the chemical nature and the postsynthetic functionalization of a framework are essential [6,7,8,9].

The inherent functionality of benzimidazole linked polymers (BILPs) and their ability to form narrow pores result in an enhanced interaction with polar guest molecules as well as high storage capacities. Thus, BILPs exhibit the highest uptakes of carbon dioxide in line with excellent selectivities compared to other flue gas participants such as nitrogen or methane, which rank them among the most promising networks for CCS applications. BILPs were first synthesized by Rabbani and El-Kaderi based on a template and catalyst-free condensation reaction of diamines with the respective aldehydes [10]. In this manner, peak carbon dioxide uptakes up to 5.34 mmol·g^−1^ (BILP-4 [11]) with a preference for CO_2_ over N_2_ of 79 and 10 for CO_2_ over CH_4_ at 273 K were observed. With a value of 113 for CO_2_/N_2_ and 17 for CO_2_/CH_4_ at 273 K, even higher selectivities were found for BILP-2 [11], although the peak uptake for carbon dioxide is only around 3.39 mmol·g^−1^, which is still remarkable in comparison to most porous polymers reported to date [11]. Consequently, further BILPs were investigated by varying the respective linker molecules to change both the morphology and the functionality of the network (Table S1, Figure S1) [12,13,14,15,16,17], making them comparable to state-of-the-art porous polymers such as PPN-6-deta [18], ALP-1 [19] or MOPI-IV [7] and metal organic frameworks such as mmen-Mg_2_(dobpdc) [20], underlining the success of this polymer class.

**Scheme 1:**
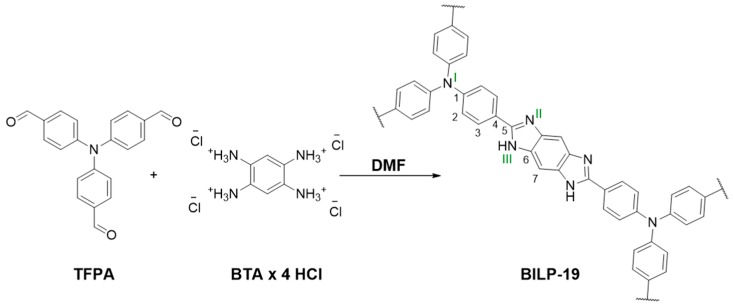
General polymerization reaction of tris(4-formylphenyl)amine (TFPA) and 1,2,4,5-benzenetetramine tetrahydrochloride (BTA∙4 HCl) to BILP-19 and its simplified structure.

Previous studies [7,21] suggest that a high uptake for carbon dioxide is closely linked to a distinct ultra-microporosity in combination with basic nitrogen containing functionalities [22,23]. Additionally we found that small, flexible linker molecules have the tendency to form narrow pore systems [22]. Therefore, we expect that the utilization of small linker molecules, which create the unique ambivalent functionality of benzimidazoles upon network formation, is promising for the synthesis of porous polymers with high affinities towards carbon dioxide [7,21]. In this respect, we report the synthesis of a new benzimidazole linked polymer (BILP-19) by a catalyst-free condensation reaction of triformylphenylamine and tetra-aminobenzene (Scheme 1). The network was fully characterized by nuclear magnetic resonance (NMR) spectroscopy (^13^C, ^15^N) and infrared spectroscopy (IR), as well as elemental (CHN) analysis, thermogravimetric analysis (TGA) and powder X-ray diffraction (PXRD). The surface area and porosity was determined from Ar and CO_2_ isotherms to explore the full spectrum of potential pore sizes. Furthermore, the gas-sorption of CO_2_, CH_4_, N_2_ and H_2_O was investigated and respective selectivities for carbon dioxide within prominent gas mixtures such as CO_2_/N_2_ and CO_2_/CH_4_ are discussed.

## 2. Results and Discussion

### 2.1. Synthesis and Characterization

**Figure 1:**
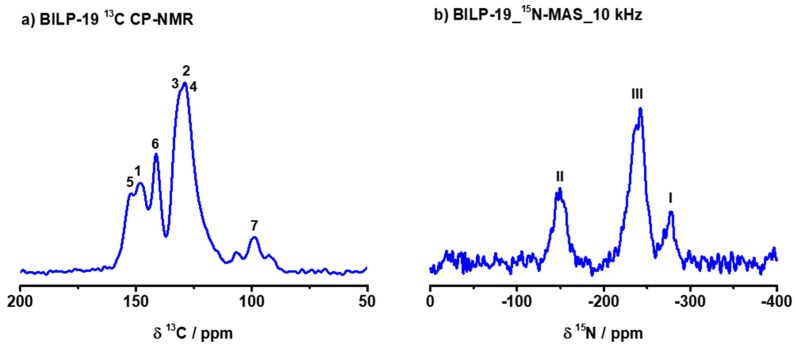
^13^C CP NMR (**a**) and ^15^N MAS NMR (**b**) spectra of BILP-19 with assignment of the signals. The spectra were measured at 12.5 kHz and 10.0 kHz, respectively. Fully observed ranges are provided in Figures S6 and S7 (Supplementary Materials).

BILP-19 was synthesized by a condensation reaction of tris(4-formylphenyl)amine (TFPA) and 1,2,4,5-benzenetetramine tetrahydrochloride (BTA∙4 HCl) as brown solid in an overall yield of 99%, following a synthesis procedure published by Rabbani and El-Kaderi (Scheme 1) [10]. The resulting network was characterized by solid–state NMR and infrared spectroscopy. In the ^13^C and ^15^N cross polarization (CP) NMR spectra, the resonances at 152 ppm (^13^C) as well as −149 and −241 ppm (^15^N) are assigned to the carbon atom C-5 and the nitrogen atoms N-II and N-III, proving the formation of the benzimidazole bridging units (Figure 1). This is underlined by infrared spectroscopy, where characteristic bands for N-H (3383 cm^−1^) and C=N (1600 cm^−1^) stretching vibrations are present (Figure S2). Additionally, the absence of the aldehyde signal, expected around 190 ppm in the ^13^C-NMR spectrum (Figure 1a) and the NH^3+^ resonances of the BTA linker in the ^15^N NMR spectrum (Figure 1b) at around −330 ppm, hint to a complete conversion of both linker molecules, anticipating a high crosslinking degree [24]. This is in line with results of the CHN analysis of BILP-19, which showed only small deviations to the calculated values (C [4.60%], H [0.09%], N [1.19%]; Table S3). Furthermore, PXRD and TGA data measured under air revealed an amorphous network structure with a thermal stability up to 494 °C (Figures S3 and S4). In the TG curve, the first 6% of weight loss are assigned to water evaporation (Figure S4). In spite of the amorphous character of BILP-19, SEM images reveal a platelet-like texture of the particles, indicating a sheet-like growth of the network (Figure S5).

### 2.2. Surface Area and Porosity

**Figure 2:**
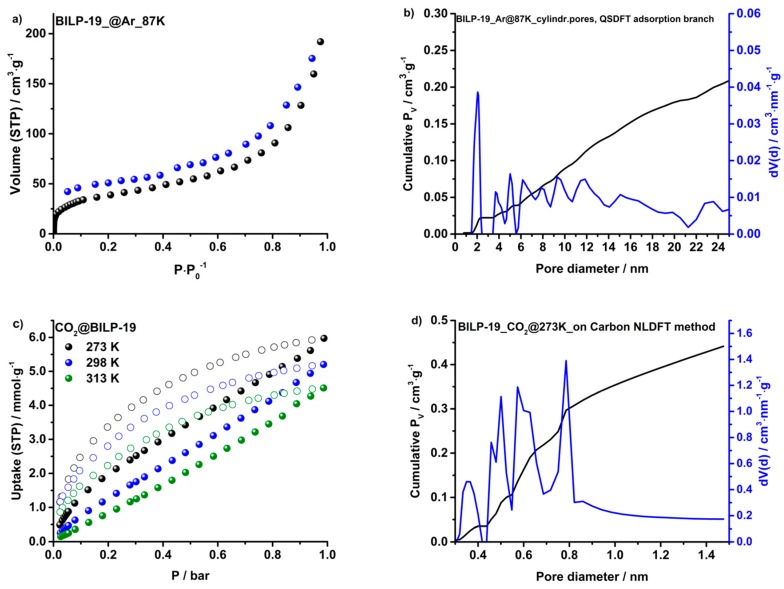
Ar adsorption isotherm (**a**) measured at 87 K and the respective, qualitative pore size distribution (**b**). CO_2_ adsorption isotherms (**c**) measured at 273 K, 298 K and 313 K. Qualitative pore size distribution calculated from the CO_2_ adsorption isotherm measured at 273 K (**d**). The gas volume and the uptake for the isotherms is given at standard temperature and pressure (STP).

The surface area of BILP-19 was calculated from Ar, N_2_ and CO_2_ adsorption isotherms measured at 87 K, 77 K and 273 K, respectively (Figure 2a, Figure S11 and Figure 2c). The Ar and N_2_ sorption isotherms exhibit a type II shape, which is typical for non- or macroporous materials [25]. Nevertheless, the steep increase at low p/p_0_ hints towards a small amount of micropores. This is in line with a type III hysteresis, typical for platelet-like particles and macropores. The *Brunauer-Emmet-Teller* (BET) surface area accessible for Ar and N_2_ was calculated to 144 and 252 m^2^·g^−1^ (Table S2). Based on a quenched solid density functional theory (QSDFT) kernel for cylindrical pores and carbon materials, the qualitative pore size distribution shows a primary mesoporous content (Figure 2b) with a small micropore contribution (p_V_mic._/p_V_tot_) of 4% and 9% (Table S2). According to the *t*-plot method, the latter is associated mainly with external surfaces (Figure S20) [26]. Due to the shape of the isotherm, we also expect the mesopores to be part of the external surface volume, created by dense packing of network platelets during sample preparation.

To probe the ultra-microporosity (pore diameter < 0.7 nm) of BILP-19, the use of carbon dioxide is advantageous due to its higher kinetic energy and thus eased penetration of narrow pores. A prerequisite is the physisorptive and reversible character of the host-guest interactions. Since no unreacted end groups like amines or aldehydes remain in the network and benzimidazole does not tend to chemical reactions with CO_2_ [12,14,27], we expect that this condition is fulfilled. The carbon dioxide adsorption isotherm revealed a remarkable surface area of 1325 m^2^·g^−1^ (Figure 2c, Table S1). Based on a nonlocal density functional theory (NLDFT) kernel for carbon materials, the calculated qualitative pore size distribution shows diameters <1 nm for the major part of the pores (Figure 2d). We expect the narrow pores to be a result of the small linker molecules, leading to short repeating units upon formation of the two-dimensional network.

### 2.3. Gas Adsorption of CO_2_, N_2_, CH_4_ and H_2_O

**Figure 3:**
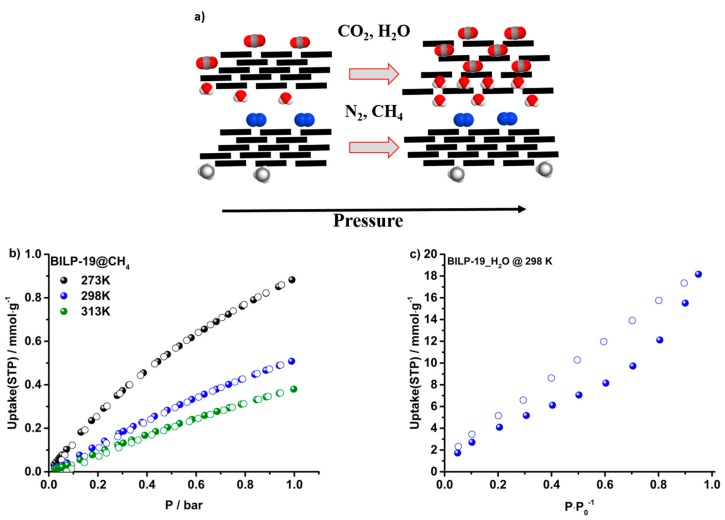
Schematic presentation of the postulated adsorption mechanism for BILP-19, where the black bars represent the polymer particles (**a**). Methane adsorption isotherms measured at 273 K, 298 K and 313 K, respectively (**b**) and a water sorption isotherm measured at 298 K (**c**). Adsorption branches are depicted by closed symbols, while open symbols represent desorption branches.

The large ultra-microporous region in combination with the poor accessibility for Ar hints to a favourable interaction with small polar guests like carbon dioxide or water. Indeed, the CO_2_ isotherms measured at 273 K, 298 K and 313 K (Figure 2c) increase steeply towards higher pressures. The desorption branch is markedly shifted towards lower *p*-values resulting in a large desorption hysteresis. We attribute this to an expansion of the interparticle space of BILP-19, where with increasing pressure (~0.1 bar) the polymer sheets expand upon carbon dioxide uptake, creating additional pore space and, therefore, better access to the inner platelets (Figure 3a). The small cavities in combination with the homogeneously distributed amount of polar groups lead to a strong interaction with carbon dioxide as well as kinetic hindrance due to long diffusion pathways, explaining the large hysteresis. Since the hysteresis does not change upon the subsequent, temperature-dependent carbon dioxide measurements, this process seems to be reversible.

Calculating thereby the amount of CO_2,_ incorporated into the network at 0.95 bar and 273 K, revealed an overall uptake of 5.97 mmol·g^−1^, which is among the highest values reported for physisorptive materials to date (Table 1). At higher temperatures, the adsorption of CO_2_ is decreased towards 5.20 mmol·g^−1^ and 4.51 mmol·g^−1^ at 298 K and 313 K, respectively. Even at these temperatures and thus more relevant conditions, the obtained values outperform most materials [3]. We attribute the superior CO_2_ affinity to be mediated by the exceptional ultra-microporosity in combination with the high density of functional groups. Both the central amino bridging-groups and the N-containing benzimidazole moieties provide preferred adsorption sites for small polar compounds like carbon dioxide or water, which are physisorptive by nature [12,14,27].

The isosteric heat of adsorption, as indicator of the type of sorption interaction was calculated from the CO_2_ isotherms at different temperatures. At zero pressure limit, values around 30 are found, which furthermore indicate a physisorptive interaction between the host material and the respective guests (Figure S8) [28]. We proposed a macroscopic swelling upon adsorption, which should change with temperature and will thus influence the isotherms. As a consequence, only the heat of adsorption for zero coverage will be reliable for our discussion [29].

For methane adsorption, however, a severely lowered uptake of 0.88 mmol·g^−1^ at 273 K was observed, which hints to a minor affinity of BILP-19 for the respective guest molecules (Table 1). Again, the uptake decreases further to 0.51 mmol·g^−1^ and 0.38 mmol·g^−1^ at 298 K and 313 K, respectively (Table 1). The low uptake values found for methane in combination with the absence of a hysteresis are a result of its size and missing affinity to the network’s functional groups, causing the disability of the molecule to penetrate the small ultra-micropores and thus to push the network layers apart (Figure 3a). In consequence, without expansion of the particles, even at higher pressures no breathing behavior is observed as for carbon dioxide and thus no additional pore space is accessible (Figure 3a). Combined with only weak interactions of the polar polymer backbone with the unpolar guests, methane could only be stored in the external interparticle voids, which contribute to only a small part of the total pore volume of BILP-19 (Table S2). Similar to methane, the affinity of the network towards nitrogen is also remarkably low, resulting in uptake values of 0.11 mmol·g^−1^ at 298 K. Again, no hysteresis was found, indicating that no breathing behavior occurs. We found low nitrogen uptakes at 298 K to be typical for polar networks in earlier work [7,22,30].

Water vapor sorption is an often neglected but important issue for the design of materials for CCS application. In this respect, especially postcombustional flue gas usually contains up to 20 vol % water vapor, which can susceptibly decrease a material’s adsorption performance when not taken into account [4]. For BILP-19, a water vapor sorption revealed an uptake of 5.17 mmol·g^−1^ at 0.3 p/p_0_ and 18.17 mmol·g^−1^ at 0.95 p/p_0_ (Figure 3c). The comparison to literature-known materials such as MOPI-IV (19.5 mmol·g^−1^) [7], AB-COF (22.9 mmol·g^−1^) [31] or TpPA-148 (24.5 mmol·g^−1^) [32] determines the network as highly hydrophilic. Based on the results of our earlier work, we once more attribute this to the high amount of ultra-micropores within the network [7]. Similar to carbon dioxide, the ultra-micropores are also penetrable for water molecules, which in combination with the polar backbone of BILP-19 leads to a high uptake. Furthermore, similar to the carbon dioxide sorption isotherms, a hysteresis occurs indicating once more a hindered desorption of polar guest molecules.

### 2.4. Selectivities over N_2_ and CH_4_

Flue gas as well as natural gas matrices are usually complex mixtures consistent of CO_2_, CH_4_, N_2_, H_2_O and other impurities such as H_2_S or NO_x_ [4,33]. Thus, additionally to high uptake values for a specific type of gas, a distinct selectivity is required. The observed behavior of BILP-19 to adsorb only minor amounts of unpolar gases, while it expresses a great affinity towards polar molecules, indicates a high selectivity towards the latter. Here we present the selectivities of BILP-19 for CO_2_ over N_2_ and CH_4_ within industrial relevant gas mixtures such as CO_2_/N_2_ (15/85) and CO_2_/CH_4_ (15/85) (Table 2). The selectivity values are calculated based on the ideal adsorbed solution theory IAST method for specific gas mixtures and initial slope of pure component-sorption isotherm calculations for a more general rating (Henry’s method) [34,35].

In this respect, for carbon dioxide over nitrogen we calculated the Henry selectivity to 123 at 298 K (Table 2). The high performance of the network is a result of its well distributed polar groups and, therefore, very low affinity towards nitrogen, especially in the low-pressure region (Figure S11). Taking the whole isotherm into account, IAST calculations for the industrial relevant gas mixture CO_2_/N_2_ (15/85) revealed a selectivity of 59, underlining its competitiveness in comparison to state-of-the-art networks (e.g. COP-1 (91) [36], MOPI-I (65) [7], TBILP-1 (62) [17], AB-COF (88) [31]. By increasing the part of carbon dioxide within the gas mixture the selectivity decreases towards 50 (Figure S21).

For carbon dioxide over methane, similar effects like for nitrogen are expected. We found a Henry selectivity of 12 at 273 K (Table 2). Interestingly, at higher temperatures the selectivity, estimated by initial slope of pure component isotherms remains similar. The same effect was found for the IAST calculations on a 15/85 CO_2_/CH_4_ mixture were the selectivity was calculated to be between 11 and 12 for 273 K, 298 K and 313 K, respectively (Supplementary Materials Section 3.2.2, Figures S22–S24). For both, nitrogen and methane, we assign the adsorption behavior primarily to a sieving effect, caused by the inaccessibility of the ultra-micropores for the respective guest. Thus, the influence of the temperature should be lowered in comparison with pure guest-functionality interactions.

## 3. Materials and Methods

### 3.1. General Information

Tris(4-formylphenyl)amine (TFPA) and 1,2,4,5-benzenetetramine tetrahydochloride (BTA∙4 HCl) were purchased from Sigma Aldrich (Steinheim, Germany). Dimethylformamide (DMF) was purchased from VWR Chemicals (Darmstadt, Germany), further dried over CaH_2_ and freshly distilled before utilization.

### 3.2. Synthesis of BILP-19

Under argon atmosphere, 0.256 g BTA × 4 HCl (9.01 × 10^−4^ mol, 2 eq.) were dissolved in 50 mL dry DMF and cooled down to −30 °C in a dry-ice/acetone bath. Afterwards a solution of 0.150 g TFPA (4.55 × 10^−4^ mol, 1 eq.) dissolved in 50 mL dry DMF was added dropwise. The reaction mixture was allowed to reach room temperature and stirred for 19 h. After flushing with air for 10 min the reaction vessel was put into an oven at 130 °C for 3 days. The resulting solid was filtered and washed with DMF, acetone and H_2_O. After soaking in 2 *M* HCl and 2 *M* NaOH for 5 min each, the material was washed again with H_2_O and acetone prior to further purification in a Soxhlet-extractor with acetone/dichloromethane (1:1) for 2 days. The brown material was dried in vacuo at 100 °C. Yield: 0.255 g (5.35 × 10^−4^ mol, 99%). ^13^C-NMR (CP-MAS, 20.0 kHz): δ [ppm] = 152 (C-5), 148 (C-1), 141 (C-6), 128_br_ (C-2, C-3, C-4), 106 (C-7) (Figure 1). ^15^N-NMR (CP-MAS, 10 kHz) = −149 (N-II), −241(N-III), −276 (N-I) (Figure 1). IR (ATR): ν [cm^−1^] = 3383, 1600, 1478, 1361, 1264, 1178, 1104, 1015, 824, 739, 683 (Figure S2).

### 3.3. Experimental Details

Argon and nitrogen sorption measurements were carried out on a Quantachrome (Odelzhausen, Germany) Autosorb-1 pore analyzer at 87 K and 298 K, respectively. The data was analyzed using the Quantachrome ASIQ v 3.0 software package. For the Ar-based isotherms, the choice of the QSDFT adsorption branch kernel for cylindrical pores in carbon-based materials depended on the calculated fitting error. The polymer was degassed under vacuum (10^−2^ kPa) at 100 °C for 12 to 16 h before starting the adsorption experiments. Selectivities and adsorption parameters were calculated using IAST and Henry methods according to the literature [34,37]. Specific surface areas, pore volumes and qualitative pore size distributions were calculated from CO_2_ isotherms at 273 K using the nonlocal density functional theory (NLDFT) model for carbon materials. The isosteric heat of adsorption was calculated using the CO_2_ adsorption isotherms at 273, 298, and 313 K. CO_2_ and CH_4_ adsorption isotherms were measured on a Quantachrome Nova surface analyzer at 273, 298, and 313 K, respectively. Water adsorption isotherms were derived from a Quantachrome Autosorb iQ at 298 K.

For infrared spectra (IR) a JASCO (Groß-Umstadt, Germany) FT/IR-6100 Fourier transform infrared spectrometer with an attenuated total reflectance (ATR) unit was used. CHN analysis was carried out at a Elementar (Langenselbold, Germany) vario EL-III with acetanilide as standard (Measured: C [65.7], H [4.2], N [17.9]. Calcd.: C [70.3], H [4.3], N [19.1]. For thermogravimetric analysis (TGA) a Mettler Toledo (Gießen, Germany) TGA/SDTA851^e^ was used. Powder X-ray diffraction (PXRD) measurements were performed on a PANalytical (Almelo, The Netherlands) X’Pert Pro diffractometer. The measurement included a region from 2 to 30° 2θ and was done with a 1/4 anti-scatter slit and Cu Kα radiation (nickel-filtered). Scanning electron microscopy (SEM) micrographs were measured with a scanning electron microscope, Zeiss (Oberkochen, Germany) LEO 1530 FESEM, equipped with a field emission cathode. All samples were drop-coated on a silicon wafer and sputtered with carbon. Energy dispersive X-ray scattering was performed with 20 kV.

All solid-state NMR MAS spectra were acquired on a Bruker (Leipzig, Germany) Avance-III HD spectrometer operating at a B_0_ field of 9.4 T. ^13^C (δ = 100.6 MHz) and ^15^N (δ = 40.6 MHz). MAS spectra were obtained with ramped cross-polarization (CP) experiments where the nutation frequency ν_nut_ on the proton channel was varied linearly from 70–100%. The samples were spun at 12.5 kHz (^13^C) and 10.0 kHz (^15^N) in a 4 mm MAS double resonance probe (Bruker). The corresponding ν_nut_ on the ^13^C channel and the contact time were adjusted to 70 kHz and 3.0 ms, respectively. On the ^15^N channel, the corresponding ν_nut_ and the contact time were adjusted to 35 kHz and 5.0 ms, respectively. Proton broadband decoupling with spinal-64 and ν_nut_ = 12.5 kHz was applied during acquisition [38]. ^13^C spectra are referenced with respect to TMS (tetramethylsilane) using the secondary standard adamantane. ^15^N spectra are referenced with respect to CH_3_NO_2_ using the secondary standard glycine.

## 4. Conclusions

We showed the successful synthesis and careful characterization of a new benzimidazole linked polymer (BILP-19) with almost complete crosslinking via a condensation reaction of triformylphenylamine and tetraaminobenzene. Despite of a negligible micro- and mostly inter-particular surface area of 144 m^2^·g^−1^ (Ar data), the network exhibits a high ultra-microporous surface area of 1325 m^2^·g^−1^. Furthermore, CO_2_ sorption isotherms revealed an exceptionally high CO_2_ uptake up to 6 mmol·g^−1^ at 273 K and water vapor sorption of 18.5 mmol·g^−1^, which is among the highest values observed for porous polymers to date. Interestingly, both water and carbon dioxide isotherms reveal a pronounced hysteresis upon desorption, while for nitrogen and methane no formation of a hysteresis was observed. Furthermore, for the latter only low uptake values (N_2_: 0.11 mmol·g^−1^ at 298 K; CH_4_: 0.88 mmol·g^−1^ at 273 K) were found. The poor affinity towards methane and nitrogen results in excellent IAST selectivities of around 12 for CO_2_/CH_4_ at 273 K, 298 K and 313 K, respectively and 60 for CO_2_/N_2_ at 298 K. We attribute the excellent adsorption and selectivity performance to a sieving effect caused by the ultra-microporosity of the network as well as of the high and well distributed amount of polar groups, in combination with a reversible expansion of the interparticle space upon adsorption. Based on our earlier work, the results once more underline the importance of a network’s ultra-microporosity on its performance of selective carbon dioxide capture. Moreover, based on its flexible packing, for BILP-19 a different way to create selectivity in two dimensional systems has been shown. Thus, the design and functionalization of ultra-microporous networks will be part of our future work.

## Figures and Tables

**Table 1:** Affinity of BILP-19 for CO_2_, CH_4_, N_2_ and H_2_O at 273 K, 298 K and 313 K taken from the respective isotherms at 0.95 bar.

T (K)	CO_2_ (mmol·g^−1^)	CH_4_ (mmol·g^−1^)	N_2_ (mmol·g^−1^)	H_2_O (mmol·g^−1^)
273	5.97	0.88	-	-
298	5.20	0.51	0.11	18.17
313	4.51	0.38	-	-

**Table 2:** Henry and IAST selectivities of BILP-19 for CO_2_ over CH_4_ and N_2_, respectively.

T/K	CO_2_/CH_4_		CO_2_/N_2_	
	Henry	IAST (15:85)	Henry	IAST (15:85)
273	12.0	11.9	-	-
298	14.0	12.3	123	59.1
313	10.3	11.3	-	-

## Supplementary Materials

All the Supplementary Materials are available online.
