# Supplementary material for: BILP-19—An Ultramicroporous Organic Network with Exceptional Carbon Dioxide Uptake
Source: Molecules. 2017 Aug 12;22(8):1343. doi: 10.3390/molecules22081343 (PMC6152267; doi:10.3390/molecules22081343)
Supplement: Supplementary file 1 [file molecules-22-01343-s001.pdf]

Supplementary Information towards:

BILP-19—An Ultramicroporous Organic Network with Exceptional Carbon Dioxide Uptake

Christoph Klumpen [1], Florian Radakovitsch [2], Andreas Jess [2] and Jürgen Senker [1,*]

[1] Inorganic Chemistry III, University of Bayreuth, Universitätsstraße 30, 95440 Bayreuth, Germany; christoph.klumpen@uni-bayreuth.de

[2] Chair of Chemical Engineering, University of Bayreuth, Universitätsstraße 30, 95440 Bayreuth, Germany; florian.radakovitsch@uni-bayreuth.de (F.R.); jess@uni-bayreuth.de (A.J.)

* Correspondence: juergen.senker@uni-bayreuth.de; Tel.: +49-(0)921-552532

Table of Content

1. General Information 2

1.1 Literature on Benzimidazole linked Polymers (BILPs) 2

2. Characterization 4

2.1 Infrared Spectroscopy (IR) 4

2.2 Powder X-ray diffraction (PXRD) 4

2.3 Thermogravimetric analysis (TGA) 5

2.4 Scanning Electron Microscopy (SEM) 6

2.5 Nuclear Magnetic Resonance (NMR) 7

2.6 Surface area and porosity 7

3. Gas Sorption and Selectivity 8

3.1 Carbon dioxide 8

3.1.1 Isosteric heat of adsorption 8

3.1.1 CO_2_Henry 9

3.1.2 CO_2_IAST 10

3.2 Nitrogen 11

3.2.1 Adsorption 11

3.2.2 N_2 Henry 12

3.2.3 N_2 IAST 12

3.3 Methane 13

3.3.1 Henry 13

3.3.2 IAST 14

3.4 Argon 16

3.4.1 T-Plot 16

3.5 Selectivities based on IAST calculations 16

3.5.1 Carbon dioxide over nitrogen 16

3.5.2 Carbon dioxide over methane 17

4. Literature 18

1. General Information

1.1 Literature on Benzimidazole linked Polymers (BILPs)

Table S1: Key values of literature known porous polybenzimidazoles. For "?" assigned networks no literature could be found.

Network	S_A (BET) / $m^2 \cdot g^{-1}$	CO_2_273K / $mmol \cdot g^{-1}$	CO_2/N_2 // 273K Henry	CO_2/CH_4 // 273 K Henry
BILP-1[1]	1172 ^{Ar}	4.27	70	10
BILP-2[2]	708 ^{Ar}	3.39	113	17
BILP-3[3]	1306 ^{Ar}	5.11	59	8
BILP-4[2]	1135 ^{Ar}	5.34	79	10
BILP-5[2]	599 ^{Ar}	2.91	95	10
BILP-6[3]	1261 ^{Ar}	4.79	63	8
BILP-7[2]	1122 ^{Ar}	4.39	62	9
BILP-8?	-	-	-	-
BILP-9?	-	-	-	-
BILP-10[4]	787 ^{Ar}	4.02	111	14
BILP-11[4]	658 ^{Ar}	3.09	103	11
BILP-12[4]	1497 ^{Ar}	5.07	56	8
BILP-13[4]	677 ^{Ar}	2.57	103	9
BILP-14?	-	-	-	-
BILP-15[5]	448 ^{Ar}	2.68	83	9
BILP-16[5]	435 ^{Ar}	2.70	53	10
BILP-15(AC) [5]	862 ^{Ar}	3.43	61	9
BILP-16(AC) [5]	643 ^{Ar}	3.45	49	9
BILP-17[6]	952 ^{Ar}	-	-	-
BILP-18[6]	947 ^{Ar}	-	-	-
BILP-101[7]	536 ^{N2}	2.43 ^{298K}	80	-
PPN-101[8]	1096 ^{N2}	5.14	199 IAST	-
TBILP-1[9]	330 ^{Ar}	117 mg/g	63	9
TBILP-2[9]	1080 ^{Ar}	228 mg/g	40	7

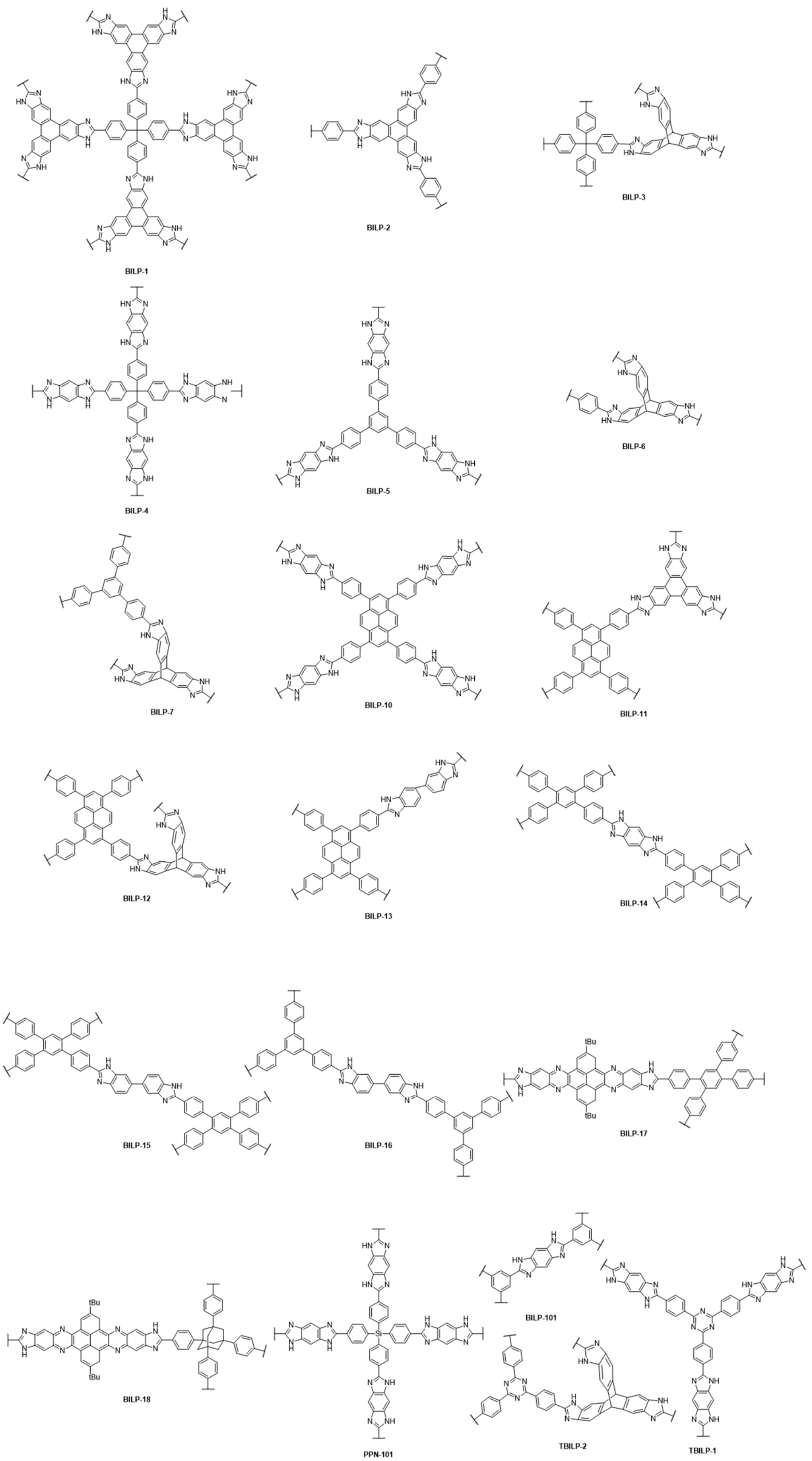

Figure S1: Schematic presentation of the benzimidazole based porous polymers compared in Table S1.

2. Characterization

2.1 Infrared Spectroscopy (IR)

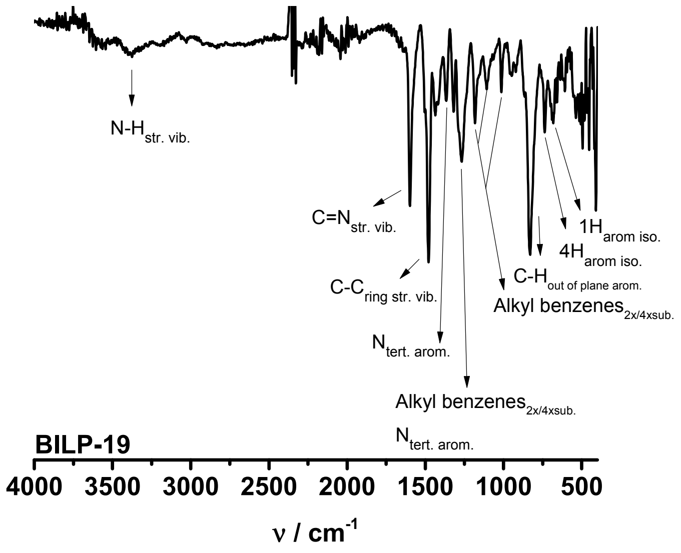

Figure S2: Infrared spectrum of BILP-19 and assignment of the most characteristic signals.

2.2 Powder X-ray diffraction (PXRD)

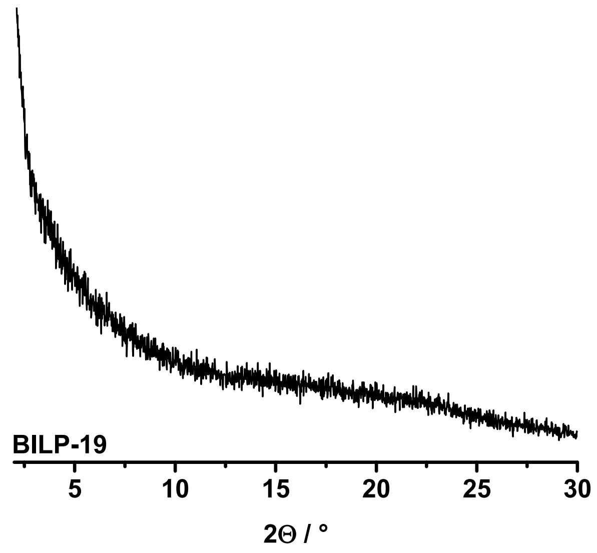

Figure S3: PXRD diffractogram of BILP-19.

2.3 Thermogravimetric analysis (TGA)

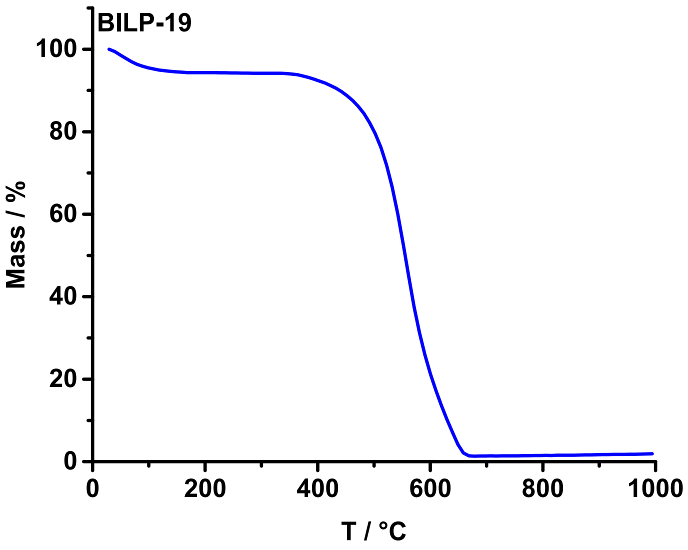

Figure S4: TGA curve of BILP-19 measured under air.

2.4 Scanning Electron Microscopy (SEM)

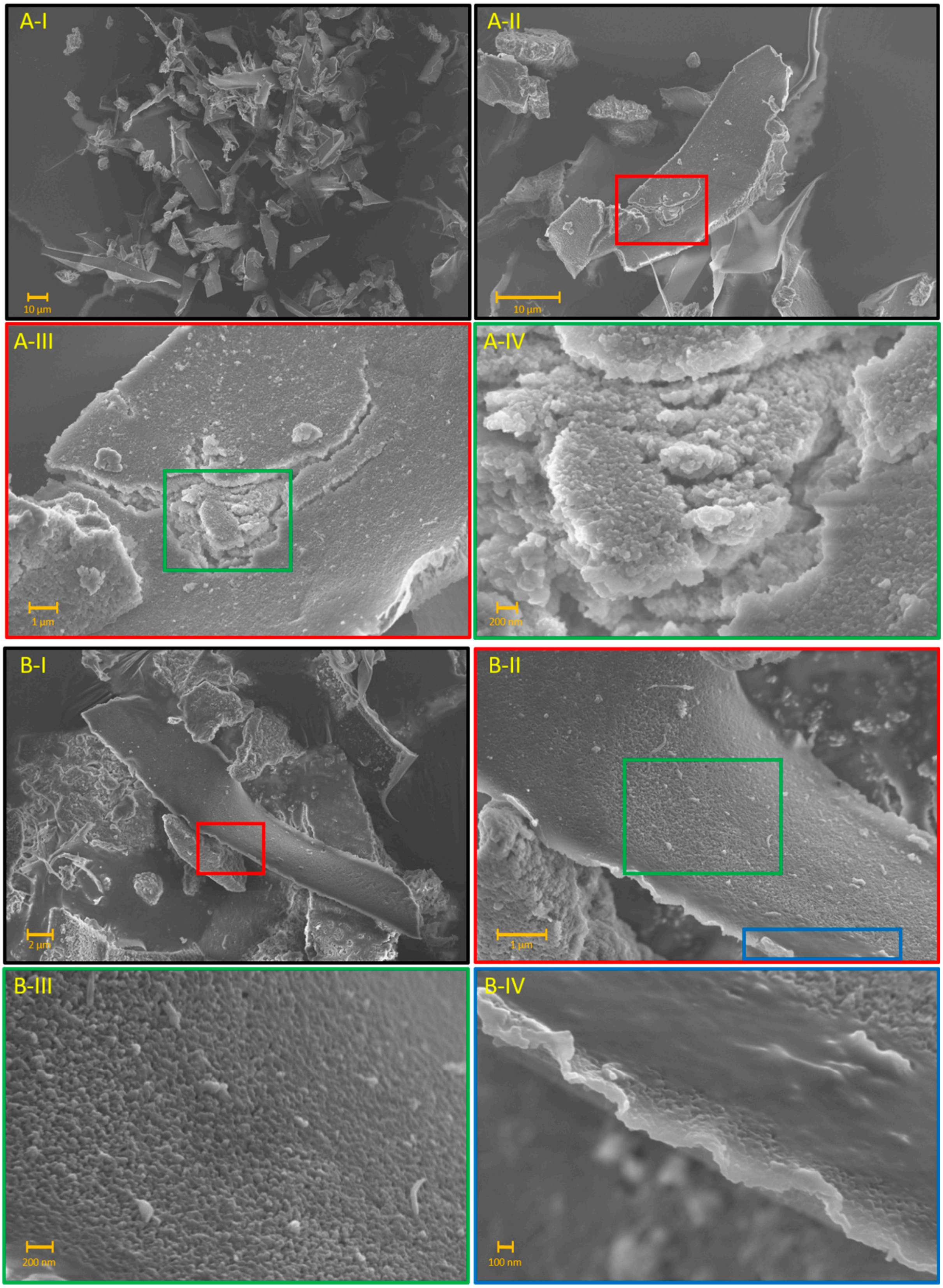

Figure S5: SEM Images of BILP-19, taken at to different positions (A, B).

2.5 Nuclear Magnetic Resonance (NMR)

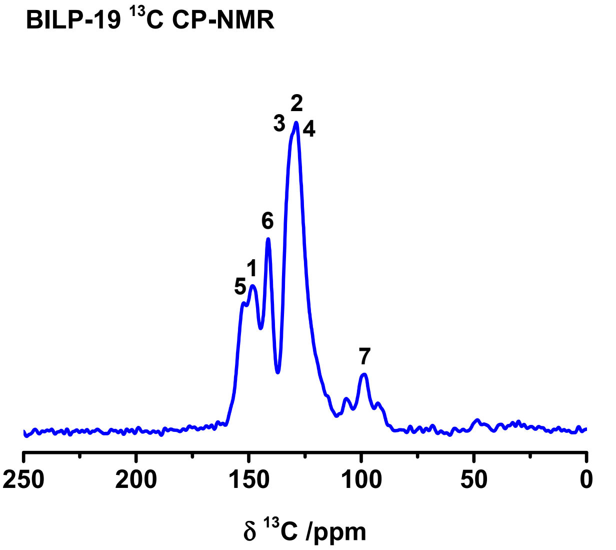

Figure S6: Complete ^{13}C MAS CP NMR spectrum of BILP-19.

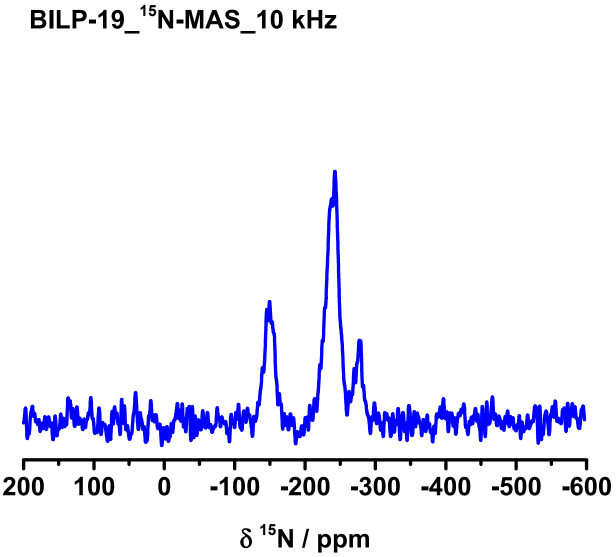

Figure S7: Complete ^{15}N CP MAS NMR spectrum of BILP-19.

2.6 Surface area and porosity

Table S2: Surface area and porosity of BILP-19 estimated from Ar, N_2 and CO_2 sorption isotherms, respectively.

Adsorbtive	S_{A_BET} / $m^2 \cdot g^{-1}$	S_{A_DFT} / $m^2 \cdot g^{-1}$	$P_{V\_tot.}$ / $cm^3 \cdot g^{-1}$	$P_{V\_mic.}$ / $cm^3 \cdot g^{-1}$	$P_{V\_mic.}$ / $P_{V\_tot.}$
Ar	144	128	0.0232	0.013	0.04
N_2	252	227	0.382	0.035	0.09
CO_2	-	1325	0.442	-	-

2.7 CHN analysis

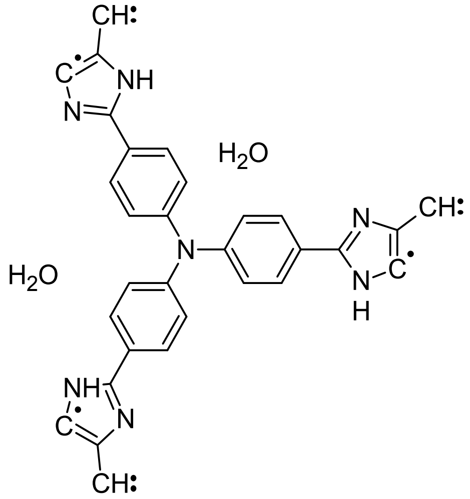

Table S3: Results of CHN analysis of BILP-19. Due to water content of 6 %, obtained by thermogravimetric analysis, two water molecules per linker were included in the theoretical calculations.

	C / %	H / %	N / %
**BILP-19·2H2Omeas.**	65.67	4.24	17.94
**BILP-19·2H2Ocalc.**	70.30	4.33	19.13
**Deviation**	4.63	0.09	1.19

3. Gas Sorption and Selectivity

3.1 Carbon dioxide

3.1.1 Isosteric heat of adsorption

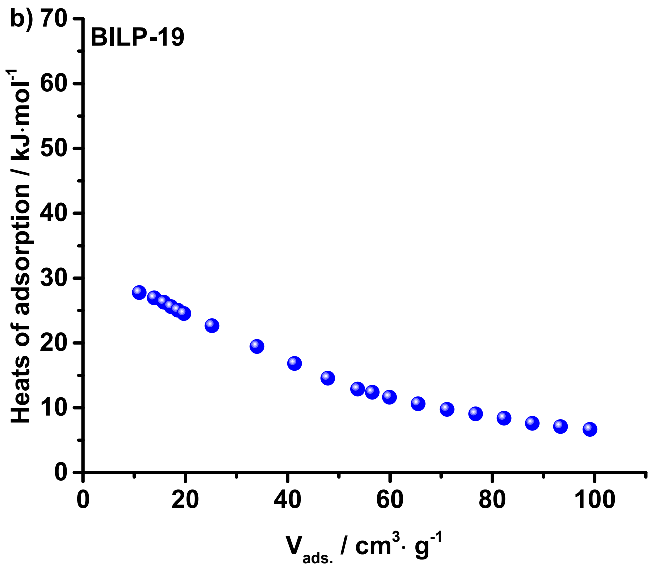

Figure S8: Isosteric heats of adsorption calculated from carbon dioxide adsorption isotherms measured at 273 K, 298 K and 313 K.

3.1.1 CO_2_Henry

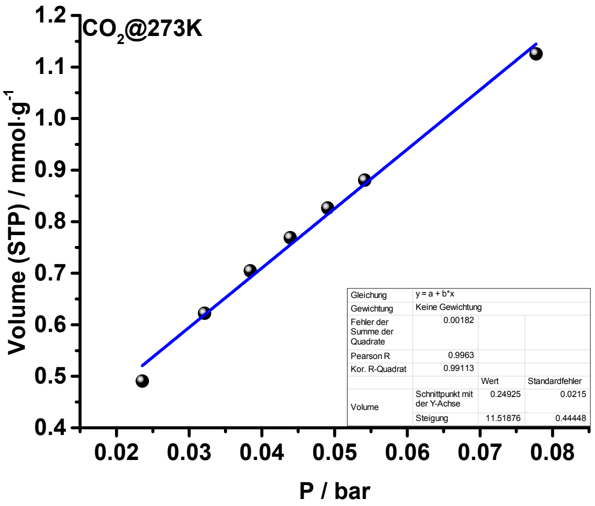

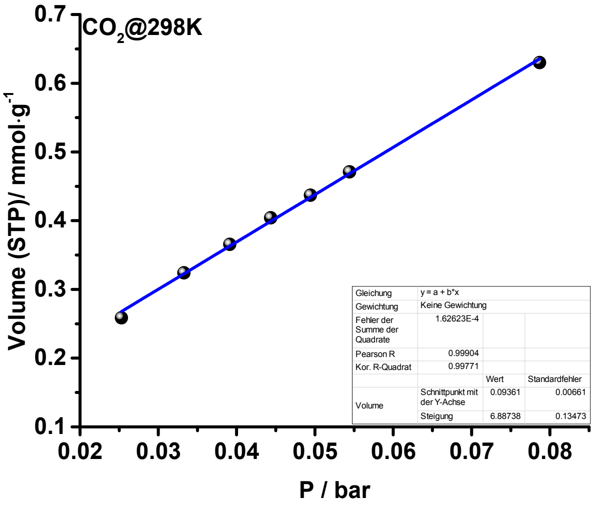

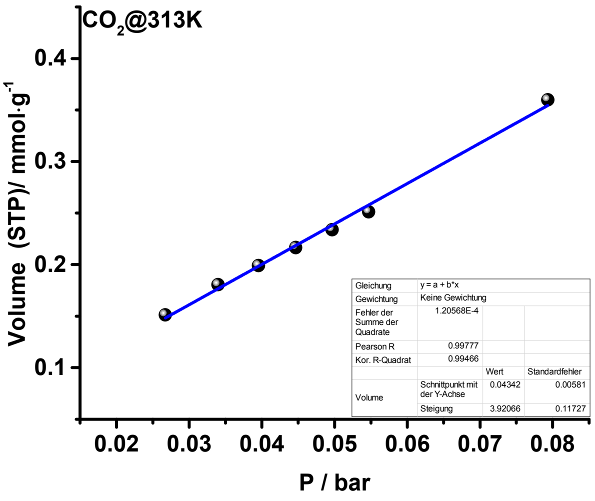

Figure S9: Fits for initial slope calculations (Henry method) of CO_2 isotherms measured at 273, 298 and 313 K.

3.1.2 CO_2_IAST

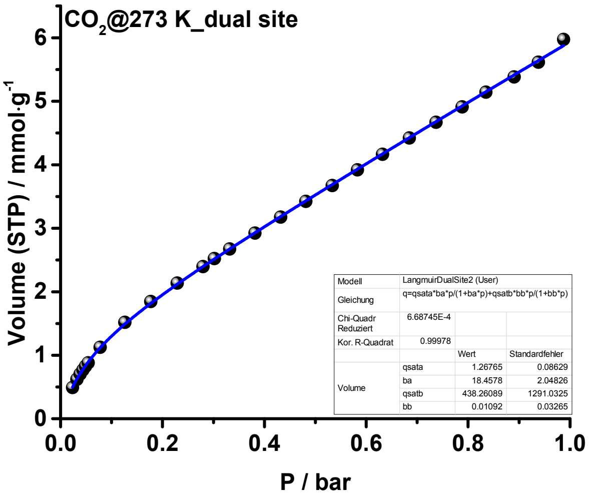

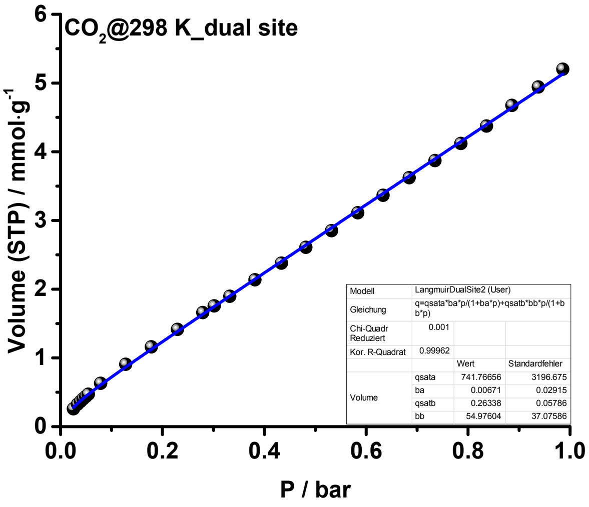

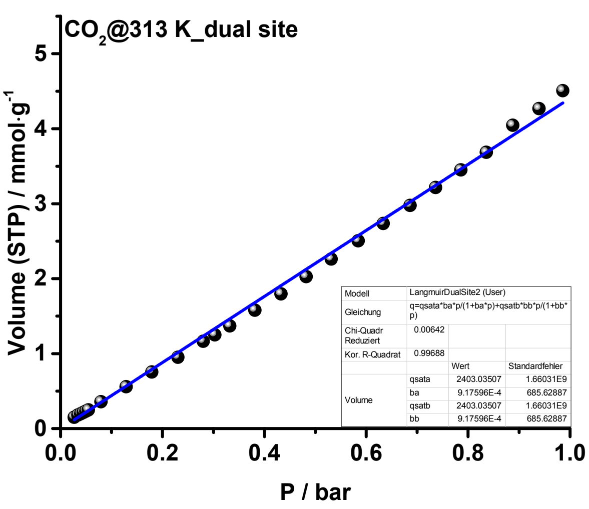

Figure S10: Langmuir dual site fits for CO_2 adsorption isotherms measured at 273, 298 and 313 K.

3.2 Nitrogen

3.2.1 Adsorption

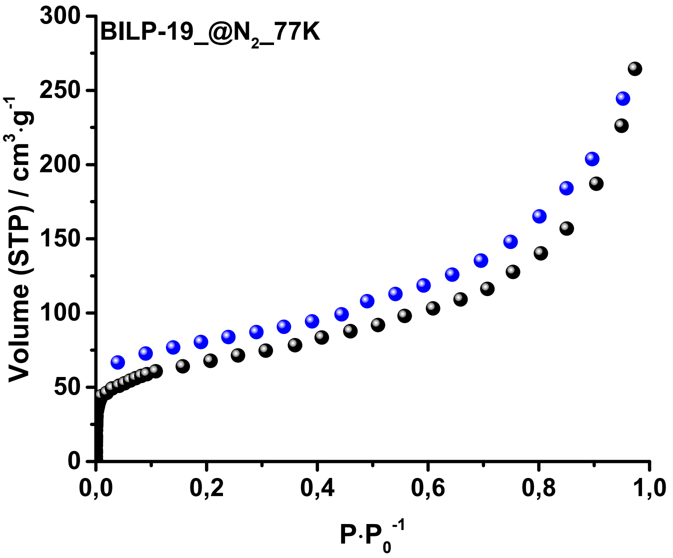

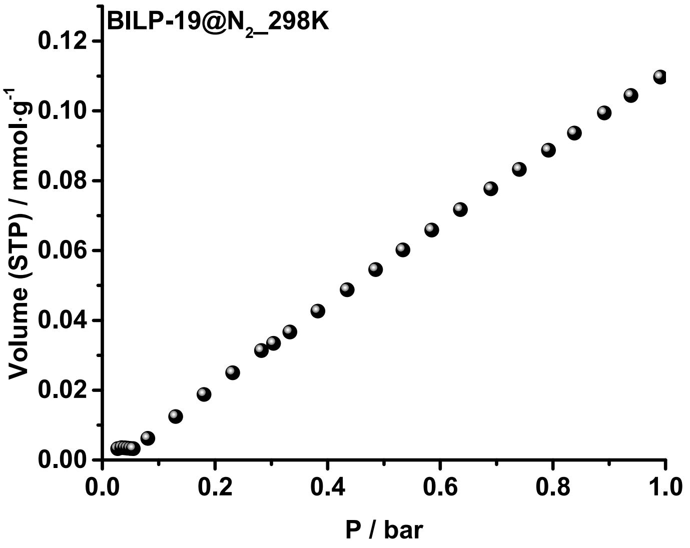

Figure S11: Nitrogen adsorption isotherm measured at 77K (top) and 298 K (bottom).

3.2.2 N_2 Henry

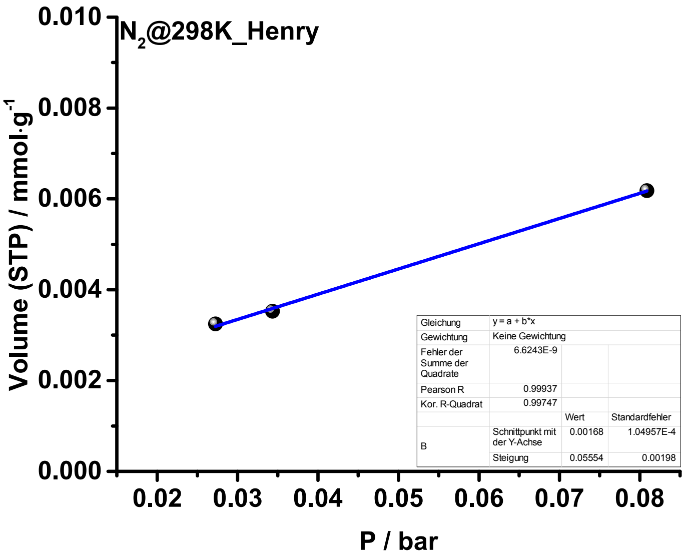

Figure S12: Initial slope of pure component fit for nitrogen adsorption at 298 K.

3.2.3 N_2 IAST

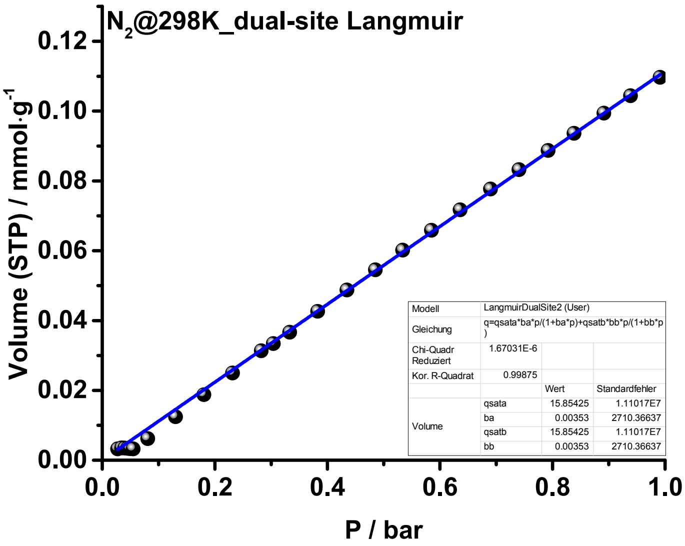

Figure S13: Langmuir dual site fit of N_2 sorption isotherms measured at 298 K.

3.3 Methane

3.3.1 Henry

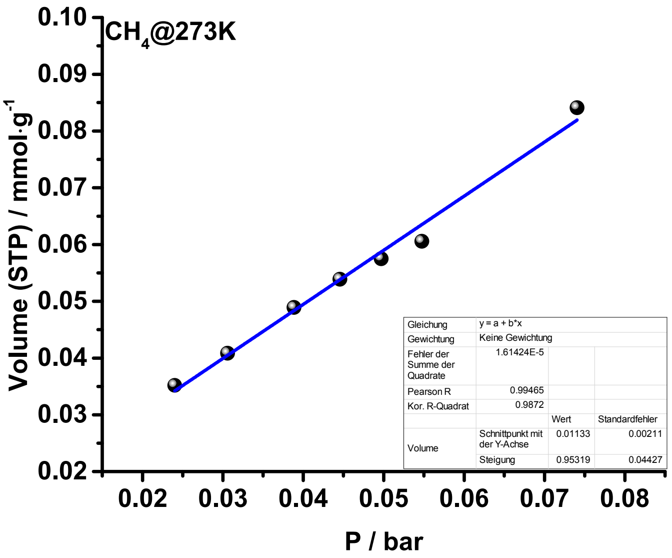

Figure S14: Initial slope of pure component adsorption fit for CH_4, measured at 273 K.

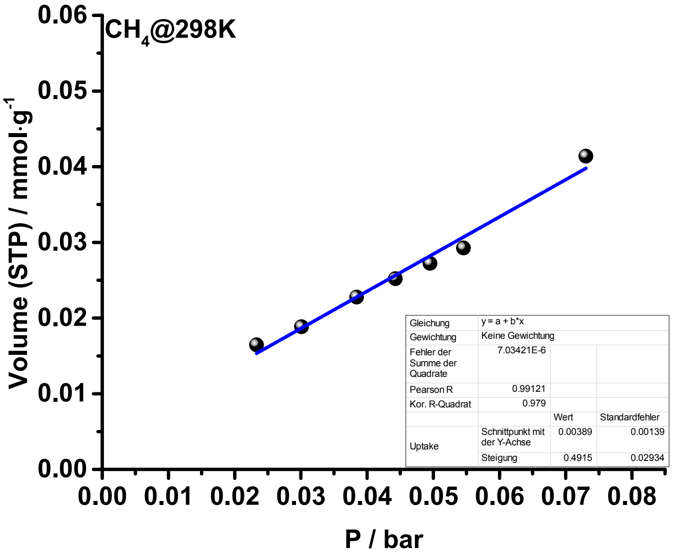

Figure S15: Initial slope of pure component adsorption fit for CH_4, measured at 298 K.

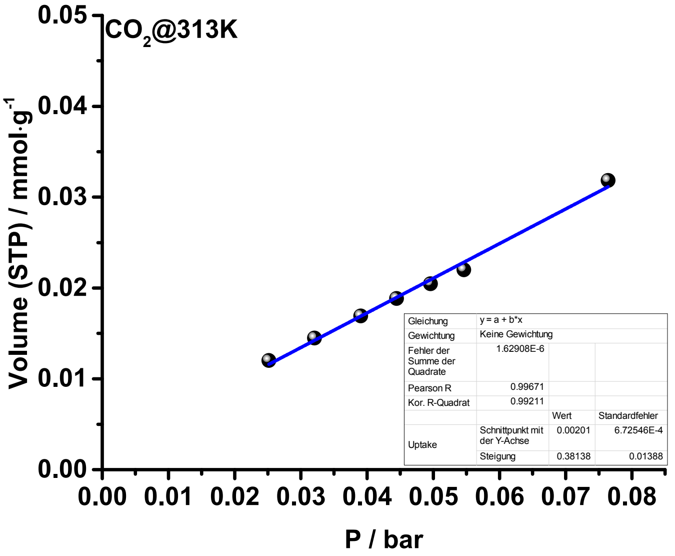

Figure S16: Initial slope of pure component adsorption fit for CH_4, measured at 313 K.

3.3.2 IAST

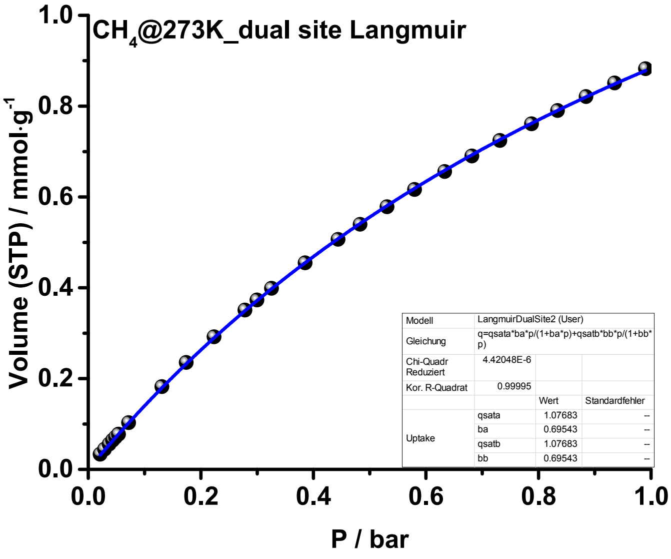

Figure S17: Langmuir dual site fit of CH_4 sorption isotherms measured at 298 K.

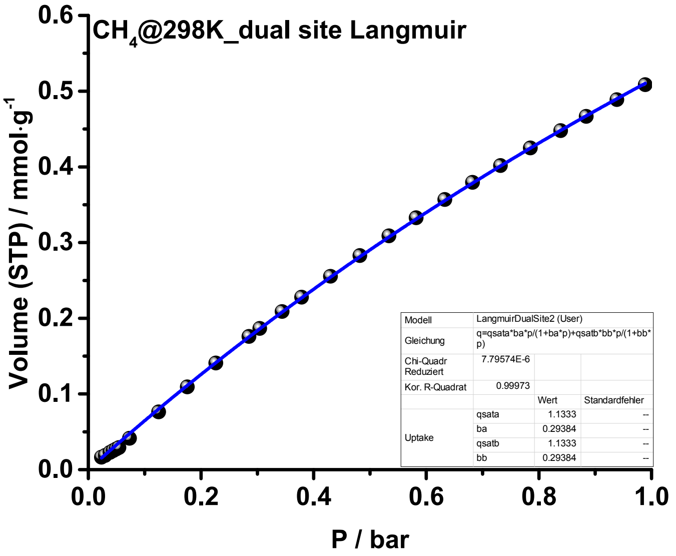

Figure S18: Langmuir dual site fit of CH_4 sorption isotherms measured at 298 K.

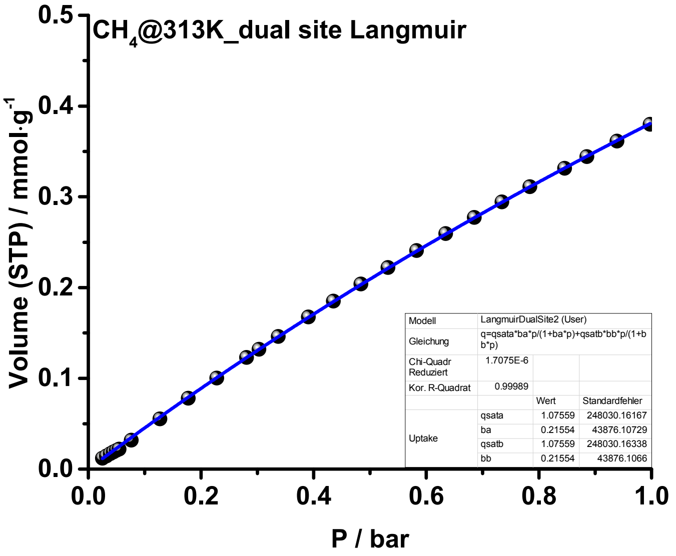

Figure S19: Langmuir dual site fit of CH_4 sorption isotherms measured at 313 K.

3.4 Argon

3.4.1 T-Plot

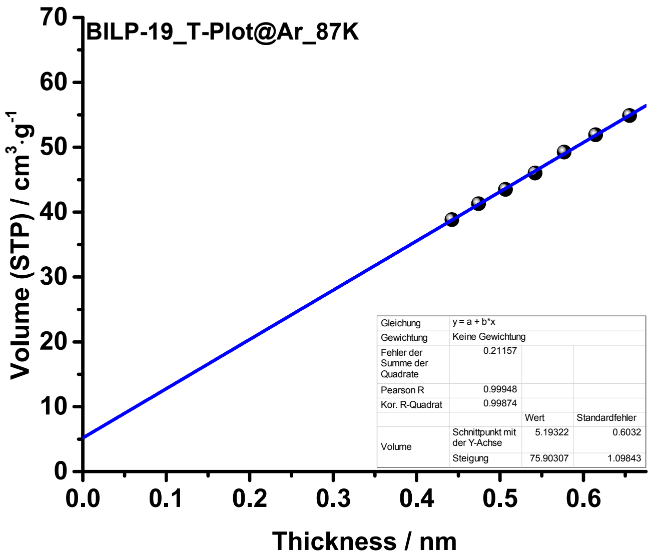

Figure S20: T-Plot applied on Ar isotherm measured at 87 K. The P/P0 range was 0.2 - 0.5.

3.5 Selectivities based on IAST calculations

3.5.1 Carbon dioxide over nitrogen

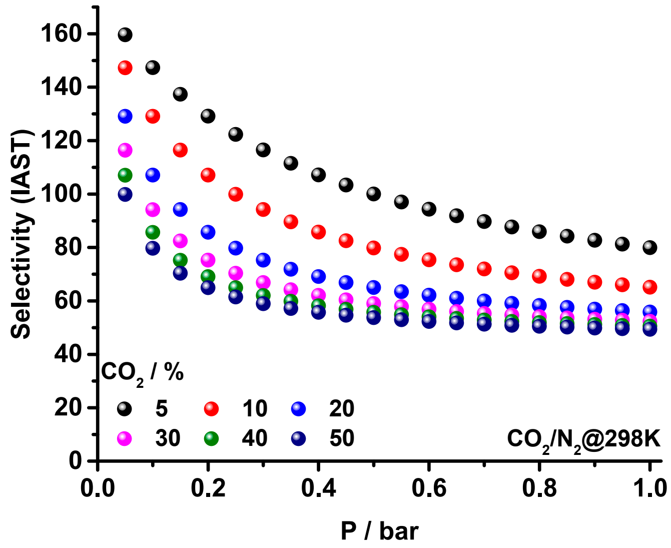

Figure S21: Selectivities of carbon dioxide over nitrogen, based on IAST calculations for different contents of carbon dioxide at 298 K.

3.5.2 Carbon dioxide over methane

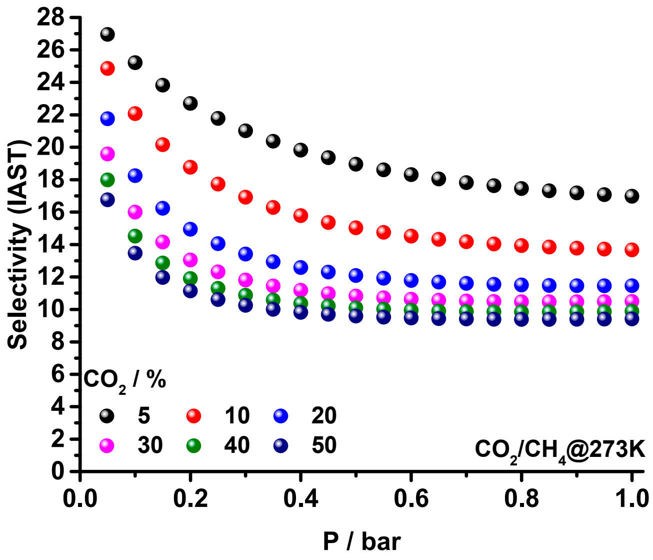

Figure S22: Selectivities of carbon dioxide over methane, based on IAST calculations for different contents of carbon dioxide at 273 K.

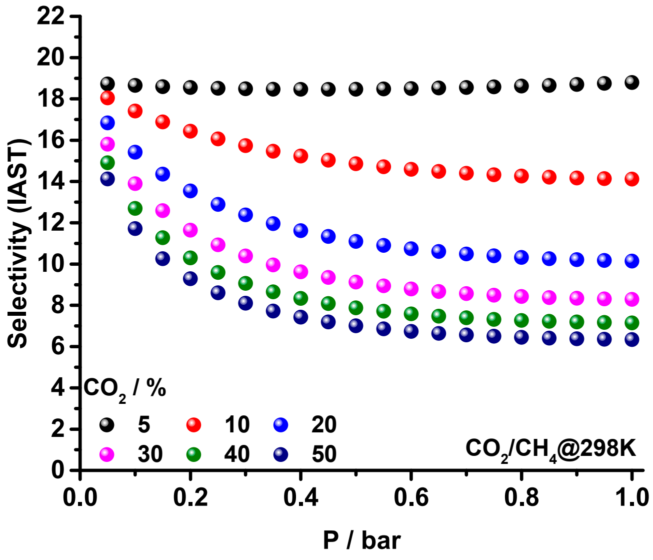

Figure S23: Selectivities of carbon dioxide over methane, based on IAST calculations for different contents of carbon dioxide at 298 K.

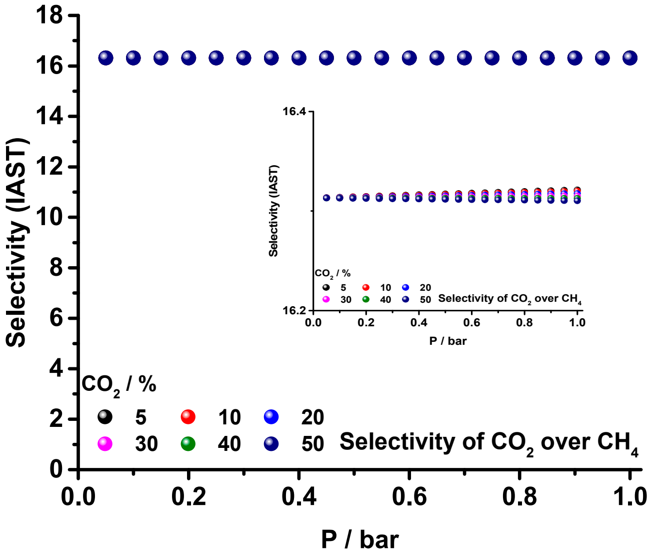

Figure S24: Selectivities of carbon dioxide over methane, based on IAST calculations for different contents of carbon dioxide at 313 K.

4. Literature

1. Rabbani, M. G.; El-Kaderi, H. M. Template-Free Synthesis of a Highly Porous Benzimidazole-Linked Polymer for CO_2 Capture and H2 Storage. *Chem. Mater.* **2011**, *23*, 1650–1653.

2. Rabbani, M. G.; El-Kaderi, H. M. Synthesis and Characterization of Porous Benzimidazole-Linked Polymers and Their Performance in Small Gas Storage and Selective Uptake. *Chem. Mater.* **2012**, *24*, 1511–1517.

3. Rabbani, M. G.; Reich, T. E.; Kassab, R. M.; Jackson, K. T.; El-Kaderi, H. M. High CO_2 uptake and selectivity by triptycene-derived benzimidazole-linked polymers. *Chem. Commun.* **2012**, *48*, 1141–1143.

4. Sekizkardes, A. K.; İslamoğlu, T.; Kahveci, Z.; El-Kaderi, H. M. Application of pyrene-derived benzimidazole-linked polymers to CO_2 separation under pressure and vacuum swing adsorption settings. *J. Mater. Chem. A* **2014**, *2*, 12492.

5. Altarawneh, S.; İslamoğlu, T.; Sekizkardes, A. K.; El-Kaderi, H. M. Effect of Acid-Catalyzed Formation Rates of Benzimidazole-Linked Polymers on Porosity and Selective CO_2 Capture from Gas Mixtures. *Environ. Sci. Technol.* **2015**, *49*, 4715–4723.

6. Altarawneh, S.; Nahar, L.; Arachchige, I. U.; El-Ballouli, A. O.; Hallal, K. M.; Kaafarani, B. R.; Rabbani, M. G.; Arvapally, R. K.; El-Kaderi, H. M. Highly porous and photoluminescent pyrene-quinoxaline-derived benzimidazole-linked polymers. *J. Mater. Chem. A* **2015**, *3*, 3006–3010.

7. Sekizkardes, A. K.; Culp, J. T.; Islamoglu, T.; Marti, A.; Hopkinson, D.; Myers, C.; El-Kaderi, H. M.; Nulwala, H. B. An ultra-microporous organic polymer for high performance carbon dioxide capture and separation. *Chem. Commun. Chem. Commun* **2015**, *51*, 13393–13396.

8. Zhang, M.; Perry, Z.; Park, J.; Zhou, H.-C. Stable benzimidazole-incorporated porous polymer network for carbon capture with high efficiency and low cost. *Polymer (Guildf).* **2014**, *55*, 335–339.

9. Sekizkardes, A. K.; Altarawneh, S.; Kahveci, Z.; Islamo⊚lu, T.; El-Kaderi, H. M. Highly selective CO_2 capture by triazine-based benzimidazole-linked polymers. *Macromolecules* **2014**, *47*, 8328–8334.
